# The effect of simulated natural environments in virtual reality and 2D video to reduce stress

**DOI:** 10.3389/fpsyg.2023.1016652

**Published:** 2023-05-12

**Authors:** Bayu Suseno, Thomas Dicky Hastjarjo

**Affiliations:** Faculty of Psychology, Gadjah Mada University, Yogyakarta, Indonesia

**Keywords:** virtual reality, 2D video, restorative, stress, simulated natural environments, physiological responses

## Abstract

*Stress* is a common problem associated with poor physical and psychological health. Exposure to the natural environment is one method for reducing stress. The real and simulated natural environments have a restorative effect on stress reduction. In contrast to the real environment, simulated natural environments, such as virtual reality and 2D video, provide safer and more controllable exposure. Several studies on the restorative effects of the natural environment in virtual reality and 2D video have been conducted. However, the difference between the two in reducing stress must be clarified. This study was conducted to determine the effect of the simulated natural environments in virtual reality and 2D video and their differences in reducing stress. This study hypothesizes that both simulated natural environments in virtual reality and 2D video can reduce stress, but there is a difference between them in reducing stress. Fifty-three subjects were divided into two experimental groups: 2D video (*n* = 28) and virtual reality (*n* = 25). The results indicated that simulated natural environments in virtual reality and 2D video reduced stress. However, there was no difference between the two groups regarding stress reduction.

## 1. Introduction

*Stress* is a common problem that has become an attribute of modern life (Kupriyanov and Zhdanov, 2014). Stress is associated with poor physical and psychological health (Toussaint et al., 2016). In the worst case, stress can cause various diseases, such as anxiety, insomnia, depression, heart disease, gastritis, and hypertension (Segerstrom and Miller, 2004).

Three theoretical models can explain the mechanism of stress. They are the stimulus model of stress, the response model of stress, and the transactional model of stress (Gaol, 2016). The stimulus model of stress explains stress as an environment that causes someone to feel depressed (Bartlett, 1998). According to Lazarus (1999), stress can be caused by the external environment and the individual’s internal state. Conversely, the stress response model explains stress as the body’s non-specific reactions to demands (Selye, 1976). Finally, the transactional stress model emphasizes the individual’s subjective evaluation of the environment as a demand for or an inability to encounter a dangerous or threatening situation. In the American Psychological Association (2015), stress is a psychological or physiological response to internal or external stressors. This study defines stress as psychological and physiological responses when a person encounters a threatening or dangerous perceived stimulus.

Stress provokes psychological and physiological responses (Kozier et al., 2017). Psychologically, stress can trigger responses, such as anxiety, fear, anger, and depression. Physiologically, stress can be explained through the general adaptation syndrome (GAS), which comprises three steps, i.e., alarm, resistance, and exhaustion (Selye, 1976). First, the body activates the physiological changes for fight-or-flight responses that increase sympathetic nervous and neuroendocrine system activity (Kozier et al., 2017) for fight-or-flight responses toward threatening stimuli. As a result, there is an increase in electrodermal activity, heart rate, respiratory rate, blood pressure, and cortisol. After the initial shock, the body attempts to overcome stressors in the resistance phase, noted by relaxation if it succeeds. Finally, exhaustion occurs when the emotional component cannot be addressed, and the body continuously generates physiological responses. In states of exhaustion, the body cannot overcome the stressor, resulting in illness.

Psychological scientists categorized stress into major life events and daily hassles (Gazzaniga et al., 2011). Major life events are any changes or strains that affect an individual’s central life. Daily hassles are minor disturbances, such as driving in heavy traffic, dealing with a difficult person, or waiting in queues. Kozier et al. (2017) divided stressors based on their origin into four types: internal, external, developmental, and situational stressors. In addition, there are types of stressors based on duration, including acute time-limited stressors, brief naturalistic stressors, stressful event sequences, chronic stressors, and distant stressors (Segerstrom and Miller, 2004). Laboratory challenges are examples of acute time-limited stressors. A brief naturalistic stressor involves confronting short-term challenges. Stressful event sequences involve an important event, such as missing someone. Chronic stressors are stressors whose duration is unknown. Distant stressors are a past traumatic experience that affects the immune system’s function continuously.

In an experimental setting, stress can be generated by manipulating stressful experiences, called acute time-limited stressors (Segerstrom and Miller, 2004). Stress can be manipulated in an experiment by giving the subjects specific tasks. For instance, the Setiatama and Kusrohmaniah (2019) employed the Sing-a-Song Stress Test (SSST), in which subjects were required to sing in front of strangers as a social-evaluative threat. SSST is a novel experimental approach that complies with ethical standards and can induce stress quickly and easily. In addition, it can control distractions resulting from sensory input and body movement (Brouwer and Hogervorst, 2014).

Many studies have been conducted about the restorative effect of a real and simulated natural environment. Previous studies have revealed a positive relationship between exposure to the real natural environment and individual health (Triguero-Mas et al., 2015; Ward Thompson et al., 2016). People who visited the forest reported feeling more comfortable, calm, and refreshed (Xiang et al., 2012; Tsunetsugu et al., 2013). Berto (2014) stated that exposure to the natural environment mediates the adverse effect of stress, reduces negative moods, and simultaneously increases positive emotions. Similarly, a forest simulated with virtual reality restored the physiological stress effect (Annerstedt et al., 2013) and improved psychological well-being (Yu et al., 2018). It was the same as the simulated natural environment in the photography slideshow (Kjellgren and Buhrkall, 2010). Video simulation of marine and forest environments also relaxed and induced changes in positive parasympathetic activities (Tsutsumi et al., 2017). In addition, the natural environmental film shown on a flat screen could have restorative effects, such as decreasing negative emotions and, vice versa, increasing positive emotions (de Kort et al., 2006). Thus, real and simulated natural environments can reduce stress (Kjellgren and Buhrkall, 2010; Hong et al., 2019).

Exposure to a simulated natural environment can be a choice to reduce stress because it is controllable and safer than the real natural environment. However, not everyone has access to the real natural environment. Because of urbanization, environmental damage, and lifestyle changes, human-nature interaction decreases quantitative and qualitative (Hartig et al., 2014). Also, not all natural environments, such as forests, are safe. A forest can pose risks to human safety. Many infectious diseases, such as the *Puumala virus (PUUV)*, *Lyme borreliosis*, *Hantavirus Cardiopulmonary Syndrome (HCPS)*, and malaria, are associated with forest, which is the preferred habitat for vectors (Aydin and Bakirci, 2007; Linard et al., 2007). Forest can also expose people to physical hazards such as forest fires, floods, droughts, landslides, and haze (Karjalainen et al., 2010).

The simulated environments commonly used are virtual reality and 2D media, for instance, 2D video. Several studies have compared the effect of virtual reality and 2D media. Compared to 2D video, virtual reality was more effective in inducing emotional and physiological responses (Ding et al., 2018), more capable of facilitating the presence effect and increasing pleasant and arousal experiences (Elsey et al., 2019), produced lower stress level (Liszio et al., 2018) and had more significant positive effects (Liszio et al., 2018; Yeo et al., 2020). Conversely, several studies found similar results between virtual reality and 2D media in reducing stress and psychological arousal (Mostajeran et al., 2021) and enhancing creativity (Palanica et al., 2019). Unfortunately, the studies comparing virtual reality and 2D media yielded inconsistent results. Therefore, this study was conducted to determine the differences between virtual reality and 2D media, i.e., 2D video, especially in reducing stress.

Unlike film or 2D video, virtual reality can provide a more immersive experience. According to Slater and Sanchez-Vives (2016), virtual reality users can experience “being there” in the virtual world through their immersive experience. The therapeutic potential is greater when the experience is more immersive (De Kort and Ijsselsteijn, 2006). Virtual reality users can also get clear, authentic experiences and a high sense of presence, which increases emotional responses and relaxation (Berto, 2014). Currently, virtual reality has been displayed through a fully immersive head-mounted display (HMD), making it possible to isolate the user’s senses from the outside world (Witmer and Singer, 1998; Higuera-Trujillo et al., 2017).

Several studies discovered the role of different participants’ geographical locations in response to environmental stimuli (Ji et al., 2000; Faggi et al., 2017). In addition, a restorative study found that the country difference significantly affected the participant’s stress recovery (Suppakittpaisarn et al., 2023). It shows that the geographical factor impacts the individual response to environmental stimuli. However, there is no restorative study, especially using a simulated environment in the Indonesian context. To the best of the researchers’ knowledge, research on the effects of the simulated environment in Indonesia has been conducted by Suyanto et al. (2017), Resibisma and Ramdhani (2020), Ramdhani et al. (2019), and Fatahillah and Hastjarjo (2021). Suyanto et al. (2017) focused on technology by developing the acrophobia application simulator to reduce acrophobia. Ramdhani et al. (2019) also Resibisma and Ramdhani (2020) focused on psychological and physiological reactions to altitude stimuli. Fatahillah and Hastjarjo (2021) focused on psychological and physiological responses to social stimuli. Regrettably, research on the effects of a simulated environment on restorative stress has never been conducted in Indonesia. By doing this study, it will be possible to determine the calming benefits of virtual reality and 2D video on stress reduction. As an outcome, this study can guide professionals who want to use a virtual environment to help clients relax.

This study was conducted to determine the effect of simulated natural environments in virtual reality and 2D video and their differences in reducing stress. Hence, this study hypothesizes that (i) both simulated natural environments in virtual reality and 2D video can reduce stress and (ii) there is a difference between simulated natural environments in virtual reality and 2D video in reducing stress levels. A simulated natural environment in virtual reality can reduce stress more than a simulated natural environment in a 2D video.

## 2. Materials and methods

### 2.1. Participants

The participants were recruited using broadcast messages and posters on social media platforms. Candidates voluntarily filled out a Google form in which the link was included in the broadcast messages and posters for enrollment and screening. The participants must meet the inclusion criteria for this research. They must be enrolled as a full-time student at universities in the Special Region of Yogyakarta, not have psychological diseases now and in the past, and have moderate to high anxiety trait scores following the categories developed by Kayikcioglu et al. (2017). There were 85 registered candidates for the study. Based on the anxiety score from the State–Trait Anxiety Inventory (STAI) trait subscale, 62 participants comprising 33 men and 29 women met the inclusion criteria. They were randomly divided into two experimental groups by range matching. First, participants were paired based on their trait anxiety scores, i.e., high and moderate. Then, participants within the same range were paired and assigned to a different group. During the implementation, nine subjects withdrew because they had other activities they had to do: six from the virtual reality group and three from the 2D video. Thus, this research’s participant was 53, comprising 25 people from the virtual reality group and 28 from the 2D video. Thirty-four participants took humanities and social science studies, 11 took engineering studies, 5 took mathematics and natural science studies, 2 took medical science studies, and 2 took agricultural studies. The virtual reality group comprised 13 men and 12 women, while the 2D video group had 16 men and 12 women. The average age of the virtual reality group was 20.7 (*SD =* 1.72), and the average of the 2D video group was 19.8 (*SD =* 1.31). There was no age difference between the virtual reality (*Mdn* = 21) and the 2D video (*Mdn* = 20) groups; *U* = 245, *p* = 0.056.

### 2.2. Stress induction

**Figure 1:**
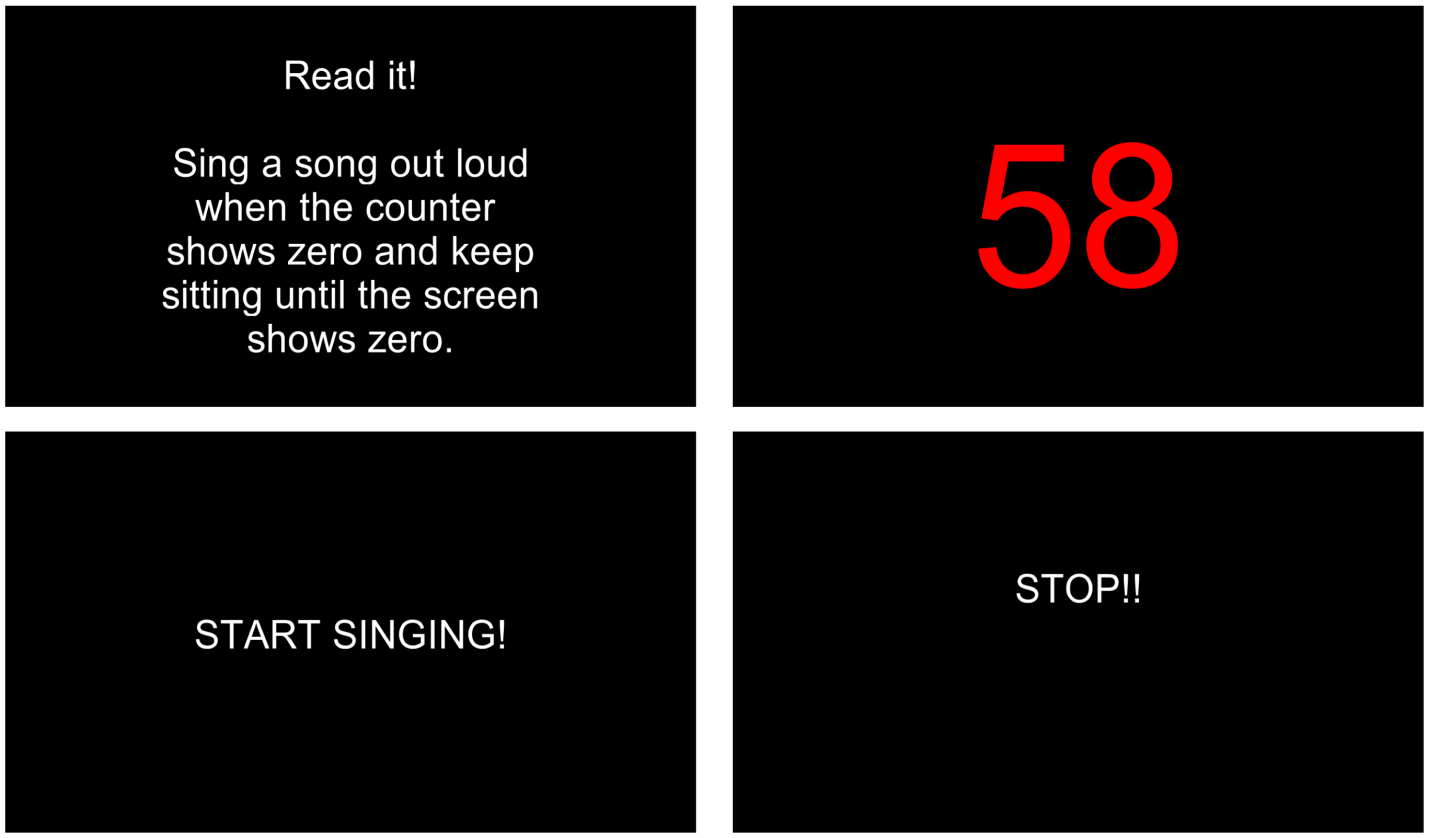
The sing-a-song stress test instructions.

The SSST developed by Brouwer and Hogervorst (2014) induced stress in this study. It is a novel, ethically compliant experimental method to induce stress quickly and easily. It can also control distractions from sensory input and body movement (Brouwer and Hogervorst, 2014). SSST used in this study was based on Setiatama and Kusrohmaniah’s (2019) study. The subjects from both groups were asked to sing any song in front of unknown people, i.e., the researcher, the assistant, and the operator, to provoke a social-evaluative threat. Social evaluative threats occur when essential aspects of oneself are, or could be, negatively judged by others (Brouwer and Hogervorst, 2014). Therefore, anticipating and watching oneself sing in front of an audience causes a solid neuroendocrine stress response (Dickerson and Kemeny, 2004) and elicits emotional stress (Harris, 2001). This study administered the SSST by instructing a task to participants in a sequence that could be seen on a flat screen (Figure 1). The first instruction, “Read it! Sing a song out loud when the counter shows zero and keep sitting still until the screen shows zero,” for 10 min. Next, the screen would show a 60-s countdown. Then, the screen showed “start singing” instructions for 15 s. Simultaneously, the subject had to sing a song until the screen showed a “stop” instruction as a sign for the participants to cease singing.

### 2.3. Simulated natural environments

**Figure 2:**
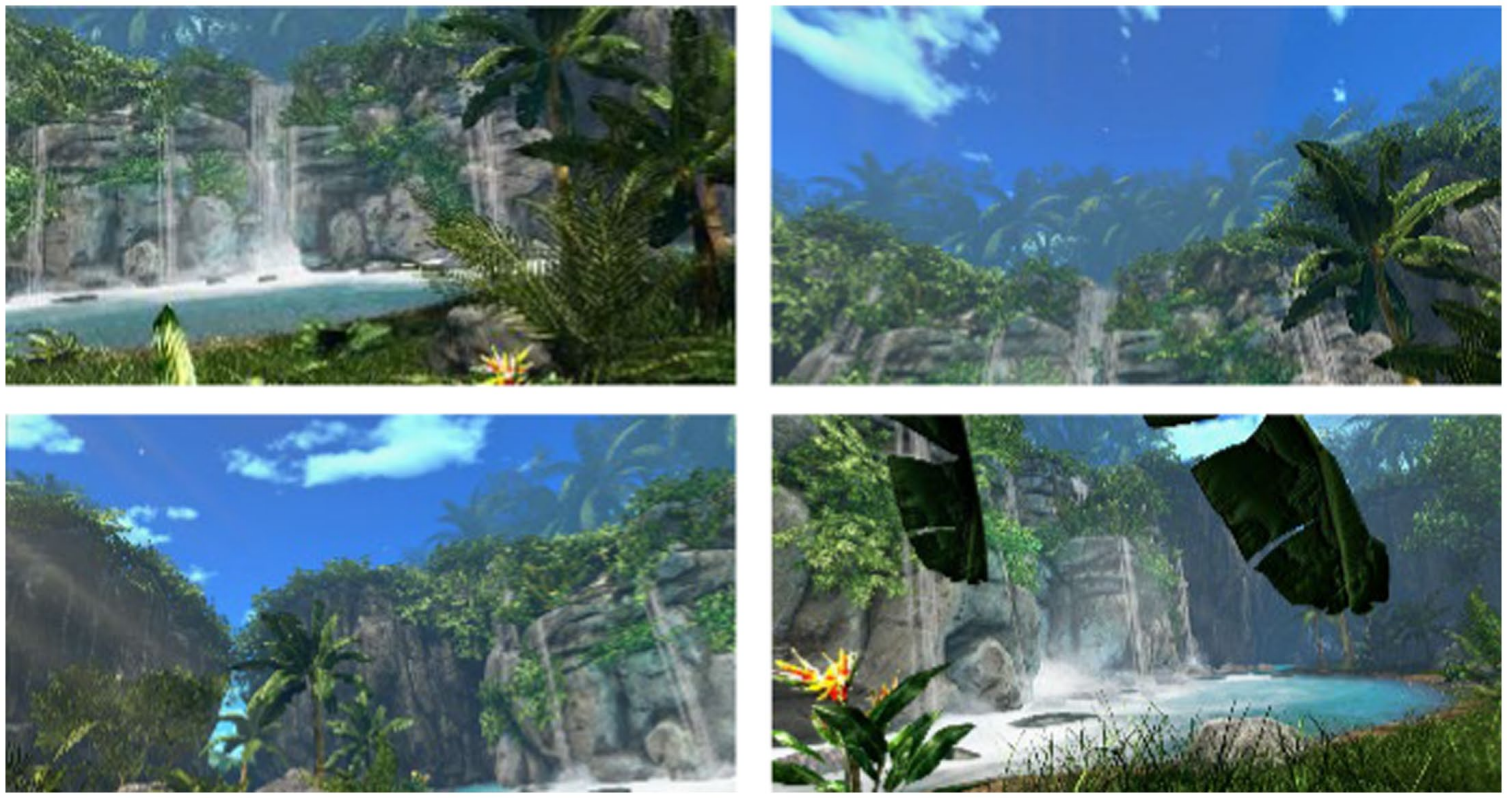
Screen captures of Nokia Bay. Reproduced with permission from Guided Meditation VR^®^, available at https://guidedmeditationvr.com/.

In this research, Nokia Bay was used as the simulated natural environment for both the virtual reality and the 2D video (Figure 2). The environment is commercially available in the Guided Meditation VR application. The application used had received permission from the developer. According to the assessment of 11 assessors who have researched the usage of virtual reality in psychology and/or been involved in the virtual reality research team of the Faculty of Psychology at UGM, the environment could generate a very high sense of presence. Witmer and Singer (1998) deemed that the effectiveness of a simulated environment is frequently linked to the sense of presence.

The virtual reality environment was shown on the HMD HTC VIVE Pro Eye, allowing subjects to explore the environment from a 3D, 360-degree point of view and equipped with a controller to move to other locations. Conversely, the 2D video environment was shown with a 22-inch flat-screen 1 m away that could only be seen from one angle. The 2D environment was produced by recording the Nokia Bay in motion mode. In both environments, the subjects could see waterfalls, trees, grass, foliage, rocks, and the sky and hear the water’s voice and birds chirping.

### 2.4. Measurement

#### 2.4.1. Stress

State–Trait Anxiety Inventory (STAI) is a scale developed by Spielberger et al. (1970) to measure an individual’s level of anxiety, which is a psychological indicator of stress (Kozier et al., 2017). In the research on the relationship between virtual reality and stress, Annerstedt et al. (2013) also applied the STAI to measure individuals’ stress levels. STAI comprised two subscales: the state and the trait subscales. Each subscale has 20 items with a choice of answers ranging from 1, “not very anxious” and “never,” to 4, “very anxious” and “always.” This research used the same scale as the research conducted by Ramdhani et al. (2019), with a coefficient alpha of 0.920 and a content validity of 0.921. The state subscale measured anxiety levels after the individual was given stress induction and environmental exposure. Meanwhile, the trait subscale was used as the participant’s screening tool.

#### 2.4.2. Self-consciousness towards body sensations

The Autonomic Perception Questionnaire (APQ) scale was developed by Mandler et al. (1958) with 24 items. The APQ scale measures self-consciousness towards bodily sensations such as sweat, temperature change, heart rate, muscle tension, respiration, digestion, and blood pressure (Seinfeld et al., 2016). According to Mandler et al. (1958), people with high self-consciousness report high levels of autonomic feedback when anxious and very high levels in stressful situations. This study used the same scale as Resibisma and Ramdhani (2020), with a coefficient alpha of 0.960 and a response range of 1–7.

#### 2.4.3. Physiological responses

The ProComp5 Infiniti biofeedback system developed by Thought Technology Ltd. was used to measure the physiological responses, i.e., the heart rate and skin conductance. The heart rate (HR) was obtained from the HR/BVP sensor on the middle finger. The electrodermal skin response was obtained from the skin conductance sensor on the index and ring fingers. All collecting physiological data were processed with BioGraph Infiniti Software V6.0.

### 2.5. Procedure

**Figure 3:**
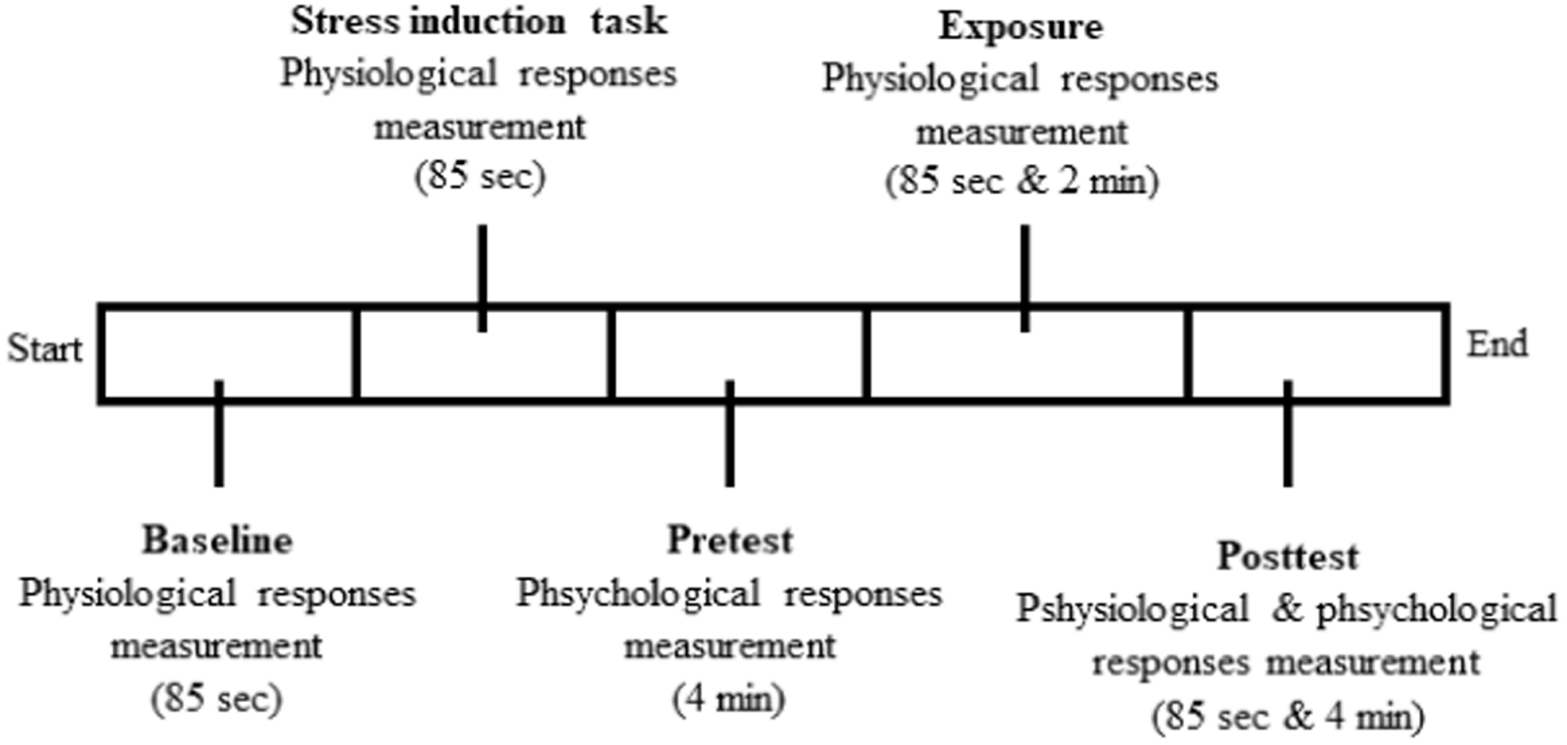
Experimental process.

The study implementation was divided into eight daily sessions, lasting about 35–45 min for each session. Each session comprised experimental processes and another procedure, including the tool installation, briefing, and break time to adjust the place and all the instruments used in this study. We defined the time based on experimental processes conducted for approximately 16 min, plus another procedure noted above took about 15–30 min. The data was retrieved in the virtual reality laboratory of the Faculty of Psychology at Gadjah Mada University. In the laboratory room, there were four people, i.e., the participant, the researcher, a computer operator, and a research assistant. The participants were asked to complete the attendance list and provide informed consent when they entered the laboratory room. Then, after agreeing to join the study, they performed a series of experimental processes (Figure 3).

First, the participants were invited to sit on the therapy chair to adjust to the laboratory for a minute. The physiological responses measurement using an HR/BVP sensor and skin conductance as a baseline was conducted for 85 s afterward. Second, the SSST as stress induction was administered to participants. Concurrently with the stress induction, physiological responses were measured for 85 s as a pre-test and manipulation check. The measurement comprised three stages, i.e., (i) 60 s during the countdown, (ii) 10 s when the instruction to sing a song out loud came up after the countdown, and (iii) 15 s when the participants were singing. Third, the participants completed the APQ scale and STAI as a psychological pre-test to determine the level of consciousness towards the body sensations and anxiety experienced during the stress induction period. Fourth, the research assistant explained to the participants what they had to do during environmental exposure. The explanation was followed by simulated environmental exposure to the participant. At the beginning of the environmental exposure, the physiological responses were measured for 85 s. After that, the participants were invited to continue exploring the simulated environment for 2 min. Fifth, the post-test measurements of physiological responses were conducted after giving environmental exposure, and post-test psychological responses were performed afterward. At the end of the session, a debriefing was given by the researcher to the participants. Participants who did not feel comfortable could see the psychologists provided by the researcher. Rewards were given to the participants as well.

#### 2.5.1. Virtual reality

The research assistant helped participants in the virtual reality group attach the HMD. HMD was used to display the environment for 3 min and 25 s. Wind stimuli coming from a fan were given to the participant as well. During the first 85 s, physiological responses were measured, so the participants were asked to remain seated while looking at the environment. The participants were then permitted to look around and use the controller to move to other places for 2 min. The participants could only move to other places, which is the application’s default, by pressing the right button. It was intended so participants could efficiently operate the controller and not move to places that blocked their view, for example, right in front of a rock. At the end of the environment displayed, the research assistant helped the participant detach the HMD.

#### 2.5.2. 2D video

The participant in the 2D video group was only seated on the therapy chair and still looked at the screen, which would display the environment. Same as the virtual reality group, the environment was displayed on the screen for 3 min and 25 s in this group. The physiological responses were also measured during the first 85 s, so participants were asked to remain seated while looking at the environment. Then, the participants continued watching the environment on the screen for 2 min. The environment was displayed in “floating motion mode” in several places, allowing the participants to see the environment flow like a river.

### 2.6. Statistical analyses

The Friedman test was used to compare stress levels across time measurement periods (within the subject). The Kruskal–Wallis test was used to compare stress levels between groups in each time measurement (between the subject). In addition, the Wilcoxon signed-rank test was used as a *post hoc* test with Holm correction to find the physiological response differences between the measurement times in each group.

## 3. Results

### 3.1. Psychological responses

#### 3.1.1. Stress

Analysis of the stress score revealed a statistically significant difference between before and after the simulated natural environment was displayed *χ^2^_F_*(1) = 40.692, *p <* 0.001, *W* = 0.768. Nevertheless, no statistically significant differences between the two groups before *H*(1) = 1.035, *p* = 0.309, *ε^2^* = 0.0199, and after *H*(1) = 1.912, *p* = 0.167, *ε^2^* = 0.0368, the simulated natural environments were displayed. Thus, both simulated natural environments in virtual reality and 2D video decreased stress levels, but there was no difference between virtual reality and 2D video in reducing stress levels (Tables 1–3).

**Table 1:** Descriptive statistics of psychological and physiological responses.

	Virtual reality			2D video		
	Mean	SD	Median	Mean	SD	Median
State STAI						
Pre-test	45.5	8.61	46.0	48.5	8.63	48.0
Post-test	31.5	5.82	32.0	35.3	8.71	33.0
APQ						
Pre-test	70.8	21.7	79.0	70.6	19.6	69.0
Post-test	55.0	17.6	52.0	58.3	19.4	52.5
Heart rate						
Baseline	87.48	12.25	87.4	90.58	90.58	90.6
Stress induction	105.70	13.16	110	108.63	17.09	110
Exposure	83.42	11.98	81.7	86.58	14.05	83.1
Post exposure	84.44	10.70	85.1	87.37	12.27	85.1
Skin conductance						
Baseline	1.29	1.18	0.870	1.44	1.49	1.07
Stress induction	2.07	2.04	1.43	2.16	2.62	1.29
Exposure	2.45	1.62	2.48	2.28	2.29	1.54
Post exposure	2.69	2.04	1.89	2.56	2.52	1.33

All the STAI and APQ data for each group could be analyzed (*n* = 25 for the virtual reality group, *n* = 28 for the 2D video group). However, one set of heart rate data in the 2D video group could not be analyzed (*n* = 25 for the virtual reality group and *n* = 27 for the 2D video group). In addition, one skin conductance data point in the virtual reality group and three in the 2D video group could not be analyzed (*n* = 24 for the virtual reality group and *n* = 25 for the 2D video group).

**Table 2:** Friedman rank test of psychological and physiological responses.

	*n*	*χ^2^*	df	*p*	*W*
State STAI					
Total	53	40.692	1	0.000^***^	0.768
Virtual reality	25	21.160	1	0.000^***^	0.846
2D Video	28	19.593	1	0.000^***^	0.700
APQ					
Total	53	30.769	1	0.000^***^	0.581
Virtual reality	25	13.500	1	0.000^***^	0.540
2D Video	28	17.286	1	0.000^***^	0.617
Heart rate					
Total	52	102.9	3	0.000^***^	0.660
Virtual reality	25	46.921	3	0.000^***^	0.626
2D Video	27	56.111	3	0.000^***^	0.693
Skin conductance					
Total	49	61.482	3	0.000^***^	0.418
Virtual reality	24	36.200	3	0.000^***^	0.503
2D Video	25	26.904	3	0.000^***^	0.359

^***^p < 0.001.

**Table 3:** Kruskal–Wallis of psychological and psychological responses.

	*χ^2^*	df	*p*	*ε^2^*
State STAI				
Pre-test	1.035	1	0.309	0.0199
Post-test	1.912	1	0.167	0.0368
APQ				
Pre-test	0.001	1	0.979	0.0000137
Postets	0.258	1	0.611	0.0049
Heart rate				
Baseline	0.128	1	0.721	0.0025
Stress induction	0.399	1	0.527	0.0078
Exposure	0.376	1	0.540	0.0073
After exposure	0.578	1	0.447	0.0113
Skin conductance				
Baseline	0.123	1	0.726	0.0025
Stress induction	0.014	1	0.904	0.0002
Exposure	0.828	1	0.363	0.0172
After exposure	0.449	1	0.503	0.0093

#### 3.1.2. Self-consciousness towards body sensations

Analysis of the self-consciousness toward body sensations score revealed a statistically significant difference between before and after the simulated natural environments were displayed *χ^2^_F_*(1) = 30.769, *p* < 0.001, *W* = 0.581. On the other hand, no statistically significant differences between the two groups before *H*(1) = 0.001, *p* = 0.979, *ε^2^* = 0.0000137, and after *H*(1) = 0.258, *p* = 0.611, *ε^2^* = 0.00496 the simulated natural environments was displayed. Thus, both simulated natural environments in virtual reality and 2D video decreased self-consciousness toward body sensations. However, there was no difference between virtual reality and 2D video in reducing self-consciousness toward body sensations (Tables 1–3).

### 3.2. Physiological responses

#### 3.2.1. Heart rate

The heart rate score analysis revealed a statistically significant main effect for time *χ^2^_F_*(3) = 102.9, *p <* 0.001, *W* = 0.660. However, there were no statistically significant differences between virtual reality and 2D video for each time measurement. Baseline condition *H*(1) = 0.128, *p* = 0.721, *ε^2^* = 0.0025, stress condition *H*(1) = 0.399, *p* = 0.527, *ε^2^* = 0.0078, exposure condition *H*(1) = 0.376, *p* = 0.540, *ε^2^* = 0.0073, and after exposure *H*(1) = 0.578, *p* = 0.447, *ε^2^* = 0.0113 (Tables 1–3).

**Figure 4:**
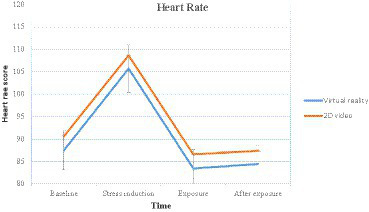
Changes in heart rate over time for the virtual reality and 2D video groups.

The results on heart rate in each time measurement (Table 4 and Figure 4) revealed that the two groups experienced significant increases from baseline to stress induction (*p* < 0.001). Conversely, the heart rate was significantly reduced while the simulated natural environments were provided (*p* < 0.001). While after the simulated natural environments had been given, there were neither significant differences in virtual reality (*p* = 0.325) nor 2D video (*p* = 0.94). Moreover, neither significant differences between exposure and after exposure in virtual reality (*p* = 0.325) nor 2D video (*p* = 0.94).

**Table 4:** *Post hoc* analysis of psychological responses using Wilcoxon signed rank test on paired sample.

	Group	Time		n	Statistic	*p*	*p* adj^*^
Heart rate	Virtual reality	1	2	25	0	0.000^***^	0.000^***^
		2	3	25	324	0.000^***^	0.000^***^
		2	4	25	324	0.000^***^	0.000^***^
		3	4	25	125	0.325	0.325
	2D video	1	2	27	0	0.000^***^	0.000^***^
		2	3	27	378	0.000^***^	0.000^***^
		2	4	27	378	0.000^***^	0.000^***^
		3	4	27	154	0.394	0.94
Skin conductance	Virtual reality	1	2	24	19.0	0.000^***^	0.001^**^
		2	3	24	89.0	0.084	0.168
		2	4	24	67.0	0.016^*^	0.048^*^
		3	4	24	98.0	0.141	0.168
	2D video	1	2	25	5.50	0.000^***^	0.000^***^
		2	3	25	109.00	0.156	0.381
		2	4	25	105.00	0.127	0.381
		3	4	25	124.00	0.312	0.381

^***^p < 0.001, ^**^p < 0.01, ^*^p < 0.05. 1 = baseline, 2 = stress induction, 3 = exposure, 4 = after exposure.

#### 3.2.2. Skin conductance

The skin conductance score analysis revealed a statistically significant main effect for time *χ^2^_F_*(3) = 61.482, *p <* 0.001, *W =* 0.418. Nevertheless, the two groups had no statistically significant differences for each time measurement. Baseline condition *H*(1) = 0.123, *p =* 0.726, *ε^2^* = 0.0025, stress condition *H*(1) = 0.014, *p =* 0.904, *ε^2^* = 0.0172, exposure condition *H*(1) = 0.828, *p =* 0.363, *ε^2^* = 0.0172, and after exposure *H*(1) = 0.449, *p =* 0.503, *ε^2^* = 0.0093. (Tables 1–3)

**Figure 5:**
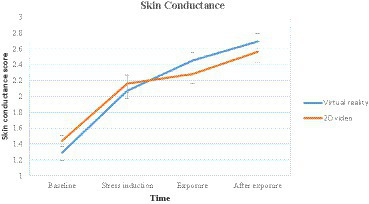
Changes in skin conductance over time for the virtual reality and 2D video groups.

Outcomes on skin conductance in each time measurement (Table 4 and Figure 5) revealed that there were significant increases from baseline to stress induction in the two groups (*p <* 0.001). When the simulated natural environments were performed, there were neither significant increments in virtual reality (*p* = 0.168) nor 2D video groups (*p* = 0.381). Interestingly, there was a significant increment in virtual reality (*p* = 0.048) but not in the 2D video group (*p* = 0.381) after the simulated natural environments had been given. Finally, there were no significant differences between exposure and after exposure in virtual reality (*p* = 0.168) or 2D video (*p* = 0.381).

## 4. Discussion

This research aims to determine the effects of simulated natural environments in virtual reality and 2D video and their differences in reducing stress. The first hypothesis is supported, as the results indicated that simulated natural environments in virtual reality and 2D video reduced emotional stress levels and self-consciousness toward body sensations. However, the result contradicts the second hypothesis about the difference between virtual reality and 2D video in reducing stress levels. No differences were obtained between virtual reality and 2D video in reducing stress and self-consciousness toward body sensations. Physiologically, both simulated natural environments in virtual reality and 2D video decreased heart rate levels that increased because of stress induction. Interestingly, skin conductance increased significantly more in the virtual reality group than in the 2D video group when the simulated natural environments were shown.

This research finding is consistent with other studies that have been conducted. Hong et al. (2019) found that, although the simulated forest in 2D video and virtual reality significantly reduced stress and heart rate, there was no difference between 2D video and virtual reality. Virtual reality aids relaxation and relieves stress, as evidenced by decreased heart rate and cortisol (Annerstedt et al., 2013). The nature of the film shown on a flat screen also increased the restorative effect of post-stress induction (De Kort and Ijsselsteijn, 2006). Nature, illustrated as a forest in the 2D video, could have a relaxing effect (Tsutsumi et al., 2017).

Possible reasons for the absence of differences can be categorized into environmental characteristics, display devices, and other factors. Environmental characteristics can be seen in the presence or absence of an avatar or virtual body and the level of interactivity in the virtual reality environment. The environmental display device included several aspects, i.e., luminance, brightness, and field of view (FoV). Other factors, such as the difference in stress levels between the virtual reality and 2D video groups, stress induction, and exposure time, could all contribute to the lack of differences.

First, there was no virtual body or avatar in the virtual reality environment used in this study. The virtual embodiment with a virtual body generates an illusion of body ownership that substitutes the user’s body (Banakou et al., 2018). An avatar’s embodiment in the virtual environment also produces a greater presence (Lugrin et al., 2015). Several studies found that a virtual body seen from the first-person perspective improved cognitive function (Osimo et al., 2015; Banakou et al., 2018) and directly affected physiological responses (Burin et al., 2020). The presence of a virtual body can also increase the effectiveness of psychotherapy applications (Gall et al., 2021). In the stress restoration context, a virtual body seen from the first-person perspective can reduce emotional and physiological stress levels (Burin et al., 2022).

Second, the interactivity of the virtual reality environment may also contribute to the absence of differences. Virtual reality is more interactive and promotes engagement in rehabilitation therapy (Choi and Paik, 2018). Participants in a virtual environment who did the activity showed a more significant overall gain in conceptual learning than those who did not (Roussou and Slater, 2020). Ferraz-Torres et al. (2022) found that interactive virtual reality produced more significant anxiety reduction and lower pain levels than passive ones in children who underwent venipuncture. The virtual reality environment in this study was used with a controller to move to other places. Still, the participants could only click a specified button to move to other places, which is the application’s default. It appeared as if the participants in the virtual reality group only explored the virtual environment by passively observing nature, similar to the 2D video group.

Third, luminance and brightness levels may produce different effects on stress reduction. Li et al. (2020) discovered that virtual reality’s brightness affected different stress levels during an intervention. This study used the default brightness level in both environments for device displays. Unlike a flat screen, the HMD blocks light from the surrounding environment. It allows the virtual environment viewed with the HMD to appear brighter. When the HMD and LCD flat screens produced the same luminance, the HMD looked brighter than the LCD flat screens (Ha et al., 2021). Another study found that the 3D virtual reality scene with an HMD was perceived as brighter than the 2D scene with a luminance camera (Lin et al., 2020). Vasylevska et al. (2019) suggested that lower brightness, particularly when combined with low brightness compensating, can be employed well when using HMD. A study also found that an environment with medium brightness significantly reduced stress levels (Li et al., 2020). It implies that using HMDs to display virtual environments needs to be adjusted to achieve more optimal results. Because this study did not address the relationship between participants’ perceptions of display brightness and stress levels, further research is needed.

Fourth, virtual reality and 2D video have a difference in FoV. The participant in the 2D video group watched the environment from one viewpoint displayed on a 22-inch flat-screen. The participant in the virtual reality group used the HTC Vive Pro HMD with a 110-degree field of view to watch the environment from a 360-degree point of view. A wide range of FoV generates a great sense of presence but contributes to higher cybersickness (Porcino et al., 2020). Conversely, reducing the FoV can reduce VR sickness (Fernandes and Feiner, 2016). A study showed that FoV restriction significantly reduced cybersickness (Teixeira and Palmisano, 2021). Cybersickness negatively impacts the user’s well-being because of the discomfort it produces (Rebenitsch and Owen, 2016). Cybersickness accounted for 98% of the variance in anxiety on a virtual roller coaster, according to a study on the relationship between cybersickness and anxiety (Bruck and Watters, 2009). Although this study did not measure the discomfort or cybersickness level, using an HMD with a bigger field of view could affect the outcome.

Factors like the stress level between virtual reality and 2D video groups, stress induction, and exposure time can also contribute to the difference. First, despite no significant differences, the stress level of the virtual reality group was lower than the 2D video group. Van den Berg et al. (2003) stated that individuals with higher stress levels are more sensitive to restoration opportunities than individuals with lower stress levels. Second, the stress that comes from the *Sing-a-Song Stress Test* does not last long. Meanwhile, the time required for participants to complete the scale is long enough. When the participants fill out the scales, their stress levels may slowly return to their original state. As a result, exposure to a simulated environment may have no effect. However, it cannot be verified because there was no real-time measurement while the scale was filled in. Third, Suppakittpaisarn et al. (2023), who did the first study comparing virtual reality exposure duration in the restoration context, found that a 5 min dose of virtual nature produced more excellent stress recovery than 1 or 15 min. They explained that the intercorrelation between time and the outcome looked like a bell-shaped pattern, which was suggested by Shanahan et al. (2016). Brown et al. (2013) discovered a significant difference in the standard deviation of respiration rate (SDRR) between viewing a nature scene and a built scene during the first 5 min, but not the second. Compared with those studies, the exposure time in this study was relatively shorter. The shorter time may not produce the optimal recovery outcome.

In line with Brouwer and Hogervorst (2014), the *Sing-a-Song Stress Test* increased heart rate and skin conductance as an indicator of stress. Lin et al. (2011) stated that stress positively correlates with the sympathetic nervous system. The increased activity of the autonomic nervous system, which is part of the sympathetic nervous system, can increase an individual’s heart rate, skin conductance, respiratory rate, and finger temperature when experiencing stress (Fink, 2016). Phelps and LeDoux (2005) stated that heart rate is commonly used to indicate stress. A systematic review study revealed a direct association between stress exposure and physiological responses, including increased heart rate (Weber et al., 2021). It means the condition of a person experiencing stress can be seen from the increased heart rate (Lin et al., 2011; De Looff et al., 2018). Therefore, a significant reduction in heart rate indicates a significant reduction in physiological stress levels.

Environments that are simulated through virtual reality and 2D video can reduce skin conductance levels (Valtchanov et al., 2010). In this study, skin conductance increased after exposure was given. However, the increase in skin conductance may not be due to the increased stress levels of the participants. Lal and Narula (2019) state that emotions can be understood by looking at two dimensions: valence and arousal. Valence describes whether something is perceived positively or negatively. In contrast, arousal describes how our bodies respond to external stimuli. For example, the emotions of pleasure and anger have the same arousal but different valences. Likewise, joyful and calm emotions have the same valence but different arousals. It means someone who feels worried and excited can show different valences and the same arousal. Hong et al. (2019) stated that the increased sympathetic nervous system activity when viewing a virtual reality forest is a positive, sympathetic activity, such as novelty and curiosity, not a harmful sympathetic activity, such as stress and pressure. Browning et al. (2020) found that participants who showed continuously increasing skin conductance levels after virtual reality exposure reported higher levels of positive affect.

A significant increase in skin conductance is only found in the virtual reality group. Chirico et al. (2017) supported the significant increase in skin conductance. Virtual reality increases skin conductance more than 2D video on a flat screen. Because virtual reality offers a more immersive experience than 2D video, it represents a significant distinction. Virtual reality users can experience being in the virtual world through immersive experiences (Slater and Sanchez-Vives, 2016). Compared to 2D video, virtual reality is more effective in inducing emotional and physiological responses (Ding et al., 2018), is more capable of facilitating the presence effect, and increases pleasant and arousal experiences (Elsey et al., 2019). Therefore, skin conductance was significantly increased only in the virtual reality group. However, it is necessary to measure immersiveness in the two environments to determine the immersiveness level of the two environments.

## 5. Limitations

This study has several limitations. First, there was no control group, so the reduction in stress levels cannot be acknowledged if the treatment is not given. Second, the resignation of subjects in each treatment group causes the number of subjects to differ. Third, the stress induction method used in this study is a novel method for producing stress that does not last long. Fourth, the exposure time in this study was relatively short.

More research is needed to discover the virtual reality and 2D video differences in reducing subjective and physiological stress. Research examining the difference between the two in reducing stress is relatively limited. Further research can be conducted by considering several things based on this study, i.e., the presence of a control group, the selection of stress induction, real-time physiological measurement during the scale’s completion, and the duration of giving environmental stimuli. Concerning the characteristics of the environment and device display, further research can add several components, such as virtual bodies or avatars, activities in the virtual environment, luminance or brightness level adjustment, and FoV restriction.

## 6. Conclusion

A natural simulated environment can be an alternative to give the restorative effect. This study showed that exposure to natural simulated environments in virtual reality and 2D video reduced emotional stress and self-consciousness toward body sensations. However, no difference was revealed between the two groups. Physiologically, both groups had a heart rate reduction, and skin conductance significantly increased only in the virtual reality group. Finally, no differences between virtual reality and 2D video were revealed regarding physiological responses in each time measurement.

## Data availability statement

The original contributions presented in the study are included in the article/supplementary material, further inquiries can be directed to the corresponding author.

## Ethics statement

The studies involving human participants were reviewed and approved by Ethics commission of Faculty of Psychology, Gadjah Mada University. The patients/participants provided their written informed consent to participate in this study.

## Author contributions

BS and TH contributed to conception and design of the study. BS organized the database, performed the statistical analysis, and wrote the first draft of the manuscript and sections of the manuscript. All authors contributed to manuscript revision, read, and approved the submitted version.

## Conflict of interest

The authors declare that the research was conducted in the absence of any commercial or financial relationships that could be construed as a potential conflict of interest.

## Publisher’s note

All claims expressed in this article are solely those of the authors and do not necessarily represent those of their affiliated organizations, or those of the publisher, the editors and the reviewers. Any product that may be evaluated in this article, or claim that may be made by its manufacturer, is not guaranteed or endorsed by the publisher.
